# Fatal Legionellosis after Water Birth, Texas, USA, 2014

**DOI:** 10.3201/eid2101.140846

**Published:** 2015-01

**Authors:** Elyse Fritschel, Kay Sanyal, Heidi Threadgill, Diana Cervantes

**Affiliations:** Texas Department of State Health Services, Arlington, Texas, USA

**Keywords:** Legionella, legionellosis, infant, water birth, bacteria, Texas

## Abstract

In 2014, a fatal infection with *Legionella pneumophila* serogroup 1 occurred in a neonate after a water birth. The death highlighted the need for infection control education, client awareness, and standardization of cleaning procedures in Texas midwife facilities.

*Legionella* species are the causative agents of legionellosis, an illness ranging in clinical presentation from a mild febrile illness known as Pontiac fever to a potentially fatal pneumonic condition termed Legionnaires’ disease (*1*). *Legionella* species are ubiquitously found in the environment, and their proliferation is supported by warm water and the presence of biofilms (*2*). Every year, 8,000–18,000 persons are hospitalized with Legionnaires’ disease in the United States (*3*). In Texas, USA, 763 confirmed or probable cases of *Legionella* infection were reported during 2008–2013 according to data from the National Electronic Disease Surveillance System (http://wwwn.cdc.gov/nndss/script/nedss.aspx). Of these case-patients, none were <1 month of age. Despite the scarcity of reported cases of legionellosis in infants, underdeveloped lungs and immune systems place infants at high risk for severe complications. The following case report summarizes the events surrounding the death of a neonate caused by *L. pneumophila* after water birth.

## The Study

In January 2014, the Texas Department of State Health Services (TDSHS) was notified of a 6-day-old infant admitted to a local pediatric hospital with loose feces, cyanosis, and respiratory failure. The infant was placed on extracorporeal membrane oxygenation because of sepsis and was prescribed ampicillin and gentamicin. Although pathogens such as *Escherichia coli*, group B *Streptococcus*, or *Listeria* were initially tested for as the suspected cause of illness, knowledge about patient exposure to a home water birth combined with symptoms of fulminant sepsis and respiratory failure led clinicians to suspect legionellosis. *Legionella* urinary antigen and PCR testing from a tracheal aspirate confirmed *L. pneumophila* serogroup 1 on day 4 of hospitalization. After 19 days of hospitalization, the infant died. The hospital confirmed *Legionella* infection as the cause of death.

Two weeks before the child’s birth, a licensed midwifery center delivered and filled a recreational-grade, jetted, soft-sided, collapsible tub with water from a private borehole well. Upon filling the tub, commercially available water purifying spa drops were added to the water. These drops are enzyme-based and do not contain chlorine. In addition, the well water had not undergone any recent filtration or chemical treatment for disinfection before use. The water circulated in the tub at ≈37°C until 2 days before the birth. At that time, the tub was drained, re-filled with well water, and left to circulate at 37°C until the delivery. The infant was born at term by spontaneous vaginal birth with no reported complications with assistance from a certified professional midwife. After the birth, the mother was transferred to a home bathtub which had been filled with well water at the time of delivery, and the infant was held there for a short time. The mother reported a healthy pregnancy and no travel during the past 12 months.

Environmental testing of the delivery tub and the private well water source was recommended by TDSHS and conducted by an Environmental *Legionella* Isolation Techniques Evaluation laboratory certified by the Centers for Disease Control and Prevention. By the time the legionellosis was reported for public health investigation, the delivery tub had been drained, disinfected, and placed in storage before being swabbed by the midwifery center. Culture isolation results from environmental swabs of the tub and well water samples did not yield *Legionella*.

Although no environmental associations were laboratory confirmed, several measures requiring remediation were discovered during the investigation. The midwifery center used a recreational jetted tub for the birth with internal tubing that can be difficult to disinfect and that is not approved for use as medical equipment. Water treatment inside the jetted tub included a non–Food and Drug Administration–approved additive with water circulating at 37°C for an extended time. Additionally, the midwifery center did not provide any written procedures for employees or clients to follow before and during the water birth.

## Conclusions

Findings from this investigation revealed a gap in the standardization and implementation of infection control practices for midwives during home water births. After reviewing available literature applicable to healthcare settings and contacting statewide midwifery and licensing agencies, the TDSHS drafted recommendations for the midwifery center associated with the reported fatal *Legionella* infection; TDSHS also distributed the recommendations to the licensing board in Texas, TDSHS regulatory officials, and professional midwifery organizations throughout Texas. The document provided guidance about the proper cleaning protocol for birthing tubs based on manufacturer recommendations and Centers for Disease Control and Prevention instruction (*4*). Recommendation was also made that recreational tubs unable to be cleaned and disinfected according these protocols should not be used. The TDSHS strongly encouraged documentation of birthing tub maintenance, including appropriate chemicals and quantities used for disinfection, as well as monitoring of pH and temperature. Additional recommendations included use of standard written procedures for employees and clients before, during, and after the water birth. These procedural documents were suggested to outline proper timing of tub filling to reduce proliferation of microorganisms, documentation of client awareness of possible risks when deviating from written procedures, and laboratory testing procedures to be followed when birthing tubs are suspected of being contaminated with *Legionella* or other pathogens.

The practice of water immersion during labor and birth has grown in popularity throughout multiple industrialized countries since the 1980s (*5*–*7*). Although midwife education about water birth exists, course curriculum and outreach are still in development. Educational and training requirements that may affect water birth infection control awareness vary by certification type, ranging from direct entry practitioners with no previous medical experience to registered practicing nurses. In a study conducted by Meyer et al. in Georgia, USA, only 30% of sampled certified nursing midwives (CNMs) had received education in their midwifery program about water birth, although most CNMs supported water birth at their facilities (*8*). Additionally, most CNMs were not moderately or severely worried about any disadvantages of water birth (*8*).

Other sporadic case reports of neonatal legionellosis after water birth have been published worldwide during the last decade (*9*–*11*). The most recent is a case of legionellosis in the United Kingdom, associated with a prefilled, whirlpool-style heated birthing tub similar to the one used in this case report (*11*). The UK environmental investigation included PCR, which yielded positive results for *L. pneumophila* of the birthing tub. Although *Legionella* culture isolation is the standard method for environmental samples, decreased isolation of the organism, especially with increased holding times, has been reported (*12*). Use of PCR in conjunction with culture isolation might increase the likelihood of detecting *Legionella* in environmental samples over culture isolation alone, but the detection of bacteria made nonviable by disinfection might lead to false positives (*13*,*14*). Environmental Legionella Isolation Techniques Evaluation certification does not yet validate for *Legionella* detection by PCR at this time. Also, limited guidance and therefore variability in sample collection techniques might greatly affect environmental isolation of *Legionella*.

Increasingly sensitive surveillance may result in additional reported cases. Continued awareness of infection potential in high-risk infants from *L. pneumophila* and other microorganisms found in potable water systems, such as *Pseudomonas aeruginosa* and *Aspergillus* spp.*,* might help ensure a safer birthing environment through community and midwifery education and enforcement of proper infection control practices.
